# Supplementary material for: Principles of resilient coding for plant ecophysiologists
Source: AoB Plants. 2021 Sep 19;13(5):plab059. doi: 10.1093/aobpla/plab059 (PMC8501907; doi:10.1093/aobpla/plab059)
Supplement: plab059_suppl_Supplementary_Materials [file plab059_suppl_supplementary_materials.pdf]

Supporting Information: Modeling photosynthesis in the {photosynthesis} package

Parameters

Part of the {photosynthesis} package allows users implement the Farquhar-von Caemmerer-Berry (FvCB; Farquhar, Caemmerer, and Berry (1980)) biochemical model of C_3 photosynthesis with temperature dependence as described in (Buckley and Diaz-Espejo 2015). A user can define leaf temperature (T_{leaf}) or provide additional parameters to calculate leaf temperature using energy balance models from the *R* package {tealeaves} (Muir 2019). The parameter inputs (Table S1) and outputs (Table S2) for modeling leaf temperature are listed in the table below, but the model details are described in Muir (2019) and in the {tealeaves} package documentation. Additional mathematical details are described in the package documentation and will be updated as necessary when new versions are released.

Temperature responses

Temperature response functions follow Bernacchi et al. (2002). For parameters that increase with leaf temperature (Γ^*, K_C, K_O, R_{d}, $V_{\text{c,max}}$, V_{tpu}), given a parameter value at 25 °C (X_{25}), the value at T_{leaf} ($X_{T\text{leaf}}$) is calculated as:

$$X_{T\text{leaf}} = X_{25} e^{\frac{E_a}{RT_{\text{ref}}} \frac{T_{\text{leaf}}-25}{T_{\text{leaf}}+273.15}}$$

where E_a is an empirical temperature response parameters and T_{ref} is 298.15 K (25 °C).

For parameters that reach their highest value at intermediate temperatures (g_{mc}, J_{max}), we use the function:

$$X_{T\mathrm{leaf}} = X_{25} e^{\frac{E_a}{RT_{\mathrm{ref}}} \frac{T_{\mathrm{leaf}}-25}{T_{\mathrm{leaf}}+273.15}} \frac{1 + e^{\frac{D_s}{R} - \frac{E_d}{RT_{\mathrm{ref}}}}}{1 + e^{\frac{D_s}{R} - \frac{E_d}{R(T_{\mathrm{leaf}}+273.15)}}}$$

where E_d and D_s are additional empirical temperature response parameters. All parameters and their default values are given in Table S3.

References

Bernacchi, Carl J, Archie R Portis, Hiromi Nakano, Susanne von Caemmerer, and Stephen P Long. 2002. "Temperature Response of Mesophyll Conductance. Implications for the Determination of Rubisco Enzyme Kinetics and for Limitations to Photosynthesis *in Vivo*." *Plant Physiology* 130 (4): 1992–8.

Buckley, Thomas N., and Antonio Diaz-Espejo. 2015. "Partitioning Changes in Photosynthetic Rate into Contributions from Different Variables: Partitioning Changes in Photosynthesis." *Plant, Cell & Environment* 38 (6): 1200–1211. https://doi.org/10.1111/pce.12459.

Farquhar, G. D., S. von Caemmerer, and J. A. Berry. 1980. "A Biochemical Model of Photosynthetic CO2 Assimilation in Leaves of C3 Species." *Planta* 149 (1): 78–90. https://doi.org/10.1007/BF00386231.

Muir, Christopher D. 2019. "Tealeaves: An R Package for Modelling Leaf Temperature Using Energy Budgets." *AoB PLANTS* 11 (6). https://doi.org/10.1093/aobpla/plz054.

Table S1: Parameter inputs for photosynthesis. Each parameter has a mathematical symbol used in the text, the R character string used in the photosynthesis package, a brief description, and the units. Baked parameters are calculated for a given leaf temperature based on their values at 25 °C and temperature response functions. For physical constants, a value is provided where applicable, though users can modify these if desired.

Symbol	R character	Description	Units
**Leaf parameters:**			
α_l	`abs_l`	absorbtivity of longwave radiation (4 - 80 μm)	none
α_s	`abs_s`	absorbtivity of shortwave radiation (0.3 - 4 μm)	none
d	`leafsize`	leaf characteristic dimension	m
Γ^*_{25}	`gamma_star25`	chloroplastic CO_2 compensation point at 25 °C	Pa
$g_{mc,25}$	`g_mc25`	mesophyll conductance to CO_2 at 25 °C	μmol m^{-2} s^{-1} Pa^{-1}
g_{sc}	`g_sc`	stomatal conductance to CO_2	μmol m^{-2} s^{-1} Pa^{-1}
g_{uc}	`g_uc`	cuticular conductance to CO_2	μmol m^{-2} s^{-1} Pa^{-1}
k_{mc}	`k_mc`	partition of g_{mc} to lower mesophyll	none
k_{sc}	`k_sc`	partition of g_{sc} to lower surface	none
k_{uc}	`k_uc`	partition of g_{uc} to lower surface	none
$K_{C,25}$	`K_C25`	Michaelis-Menten constant for carboxylation at 25 °C	μmol mol^{-1}
$K_{O,25}$	`K_O25`	Michaelis-Menten constant for oxygenation at 25 °C	μmol mol^{-1}
ϕ_J	`phi_J`	initial slope of the response of J to PPFD	none
$R_{d,25}$	`R_d25`	nonphotorespiratory CO_2 release at 25 °C	μmol CO_2 m^{-2} s^{-1}
θ_J	`theta_J`	curvature factor for light-response curve	none
T_{leaf}	`T_leaf`	leaf temperature	K
$V_{c,max,25}$	`V_cmax25`	maximum rate of carboxylation at 25 °C	μmol CO2 m^{-2} s^{-1}
$V_{tpu,25}$	`V_tpu25`	rate of triose phosphate utilization at 25 °C	μmol CO2 m^{-2} s^{-1}
**Baked parameters:**			
g_{mc}	`g_mc`	mesophyll conductance to CO_2 at T_{leaf}	μmol m^{-2} s^{-1} Pa^{-1}
K_C	`K_C`	Michaelis-Menten constant for carboxylation at T_{leaf}	μmol mol^{-1}
K_O	`K_O`	Michaelis-Menten constant for oxygenation at T_{leaf}	μmol mol^{-1}
R_d	`R_d`	nonphotorespiratory CO_2 release at T_{leaf}	μmol CO_2 m^{-2} s^{-1}
$V_{c,max}$	`V_cmax`	maximum rate of carboxylation at T_{leaf}	μmol CO_2 m^{-2} s^{-1}
V_{tpu}	`V_tpu`	rate of triose phosphate utilisation at T_{leaf}	μmol CO_2 m^{-2} s^{-1}
**Environmental parameters:**			
C_{air}	`C_air`	atmospheric CO_2 concentration	Pa
E_q	`E_q`	energy per mole quanta	kJ mol^{-2}
f_{PAR}	`f_par`	fraction of incoming shortwave radiation that is photosynthetically active radiation (PAR)	none
O	`O`	atmospheric O_2 concentration	kPa
P	`P`	atmospheric pressure	kPa
r	`r`	reflectance for short-wave irradiance (albedo)	none
RH	`RH`	relative humidity	none
PPFD	`PPFD`	photosynthetic photon flux density	μmol quanta m^{-2} m^{-1}
T_{air}	`T_air`	air temperature	K
u	`wind`	wind speed	m s^{-1}
**Physical constants:**			
a, b, c, d	`a, b, c, d`	coefficients for calculating Nu and Sh numbers	none
c_p	`c_p`	heat capacity of air	1.01 J g^{-1} K^{-1}
$D_{c,0}$	`D_c0`	diffusion coefficient for CO_2 in air at 0 °C	12.9×10^{-6} m^2 s^{-1}
$D_{h,0}$	`D_h0`	diffusion coefficient for heat in air at 0 °C	19.0×10^{-6} m^2 s^{-1}
$D_{m,0}$	`D_m0`	diffusion coefficient for momentum in air at 0 °C	13.3×10^{-6} m^2 s^{-1}
$D_{w,0}$	`D_w0`	diffusion coefficient for water vapour in air at 0 °C	21.2×10^{-6} m^2 s^{-1}
ϵ	`epsilon`	ratio of water to air molar masses	0.622
eT	`eT`	exponent for temperature dependence of diffusion	1.75
G	`G`	gravitational acceleration	9.8 m s^{-2}
$\bar{R}$	`R`	ideal gas constant	8.3144598 J mol^{-1} K^{-1}
R_{air}	`R_air`	specific gas constant for dry air	287.058 J kg^{-1} K^{-1}
σ	`s`	Stefan-Boltzmann constant	5.67×10^{-8} W m^{-2} K^{-4}

Table S2: Parameter outputs for photosynthesis. Each parameter has a mathematical symbol used in the text, the *R* character string used in the photosynthesis package, a brief description, and the units.

Symbol	R character	Description	Units
A	`A`	photosynthetic rate at C_{chl}	$\mu mol\ CO_2\ m^{-2}\ s^{-1}$
C_{chl}	`C_chl`	chloroplastic CO_2 concentration where A_{supply} intersects A_{demand}	Pa
g_{tc}	`g_tc`	total conductance to CO_2 at T_{leaf}	$\mu mol\ m^{-2}\ s^{-1}\ Pa^{-1}$

Table S3: Empirical temperature response parameters for photosynthesis. Each parameter has a mathematical symbol used in the text, the *R* character string used in the photosynthesis package, and the units with default value that can be changed by users.

Symbol	R character	Units
$D_{s,gmc}$	`Ds_gmc`	487.29 $J\ mol^{-1}\ K^{-1}$
$D_{s,J\text{max}}$	`Ds_Jmax`	388.04 $J\ mol^{-1}\ K^{-1}$
$E_{a,\Gamma*}$	`Ea_gammastar`	24459.97 $J\ mol^{-1}$
$E_{a,gmc}$	`Ea_gmc`	68901.56 $J\ mol^{-1}$
$E_{a,J\text{max}}$	`Ea_Jmax`	56095.18 $J\ mol^{-1}$
$E_{a,KC}$	`Ea_KC`	80989.78 $J\ mol^{-1}$
$E_{a,KO}$	`Ea_KO`	23719.97 $J\ mol^{-1}$
$E_{a,R\text{d}}$	`Ea_Rd`	40446.75 $J\ mol^{-1}$
$E_{a,V\text{cmax}}$	`Ea_Vcmax`	52245.78 $J\ mol^{-1}$
$E_{d,gmc}$	`Ed_gmc`	148788.56 $J\ mol^{-1}$
$E_{d,J\text{max}}$	`Ed_Jmax`	121244.79 $J\ mol^{-1}$
