# Supplementary material for: Principles of resilient coding for plant ecophysiologists
Source: AoB Plants. 2021 Sep 19;13(5):plab059. doi: 10.1093/aobpla/plab059 (PMC8501907; doi:10.1093/aobpla/plab059)
Supplement: plab059_suppl_Supplementary_Notes [file plab059_suppl_supplementary_notes.pdf]

Supporting Information: Benchmark comparison of `plantecophys::fitaci` and `photosynthesis::fit_aci_response`

In this section, we compare parameter estimates of the Farquhar-von Caemmerer-Berry C_3 biochemical model (Farquhar, Caemmerer, and Berry 1980) using the default settings for the `fitaci` function from {plantecophys} version 1.4.6 and the `fit_aci_response` function from {photosynthesis} version 2.0.2. In this and future benchmark comparisons we will use (nearly) default settings likely to resemble common user experience. The default model assumptions are not identical, so this should reveal a typical amount of discrepancy between packages.

We compared the fit of $A - C_i$ curves reported from four species by Slot and Winter (2017). We downloaded the data from Kumarathunge et al. (2019) and extracted curves. If you have an Internet connection, you can download the data from an online repository:

```
global_aci = readr::read_csv("https://figshare.com/ndownloader/files/13449305")
```

We extracted appropriate curves and prepared them for fitting with the following code:

```
library(dplyr)
library(tidyr)


slot_winter_2017 = global_aci |>
  filter(
    Reference == "Slot and Winter (2017b) and Slot and Winter (2017a)",
    Species %in% c("Calophyllum longifolium", "Ficus insipida",
```

```r
                    "Garcinia madruno", "Lagerstroemia speciosa"),
    !is.na(Curve_Id),
    !(Species == "Ficus insipida" & Curve_Id == 117),
    !(Species == "Garcinia madruno" & Curve_Id == 79.1)
  ) |>
  unite(id, Species, Curve_Id, remove = FALSE) |>
  mutate(T_leaf = Tleaf + 273.15) |>
  as.data.frame()
```

We fit $A - C_i$ curves assuming no TPU limitation in both packages with the following code:

```r
# Fit with {plantecophys}
# Tcorrect = FALSE for comparability with {photosynthesis} output
fit1 = fitacis(slot_winter_2017, "id", Tcorrect = FALSE)

# Fit with {photosynthesis}
# fitTPU = FALSE and useg_mc = FALSE for comparability with {plantecophys} output
fit2 = fit_many(
  data = slot_winter_2017,
  varnames = list(
    A_net = "Photo",
    T_leaf = "T_leaf",
    C_i = "Ci",
    PPFD = "PARi"
  ),
  funct = fit_aci_response,
  group = "id",
  fitTPU = FALSE,
  useg_mc = FALSE
)
```

The estimated parameter values are very similar between packages.

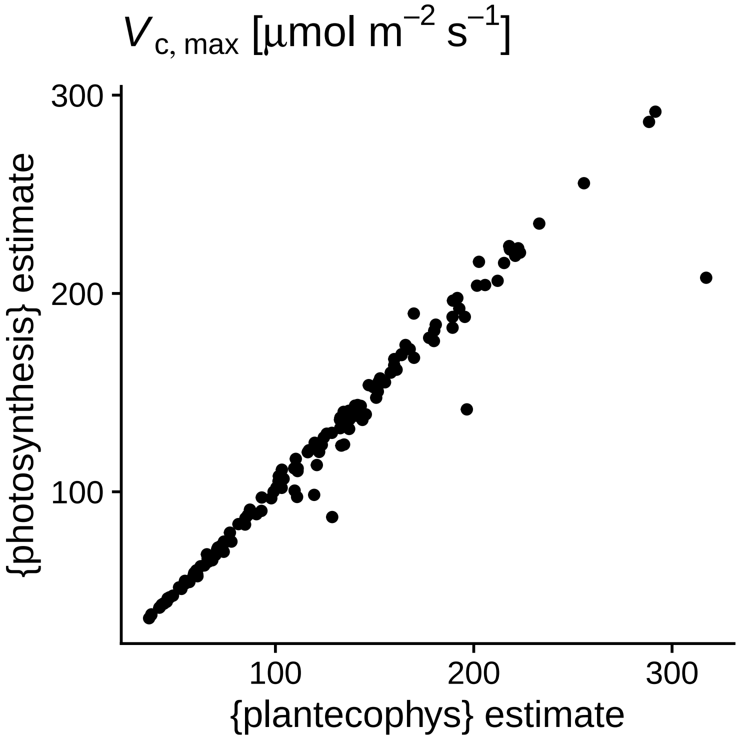
$V_{c, max}$ [μmol m^{-2} s^{-1}]
{photosynthesis} estimate
{plantecophys} estimate
100
200
300

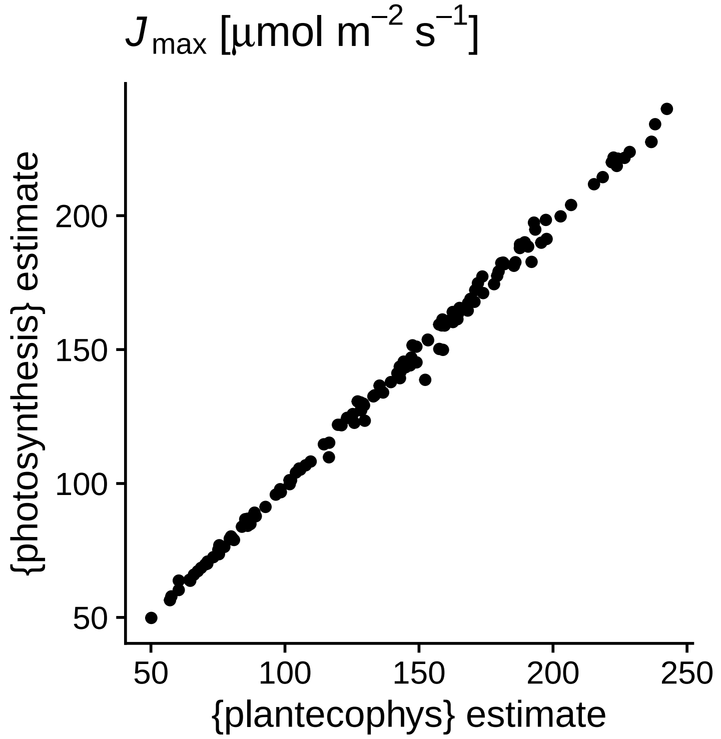
J_{max} [μmol m^{-2} s^{-1}]
{photosynthesis} estimate
{plantecophys} estimate
50
100
150
200
250

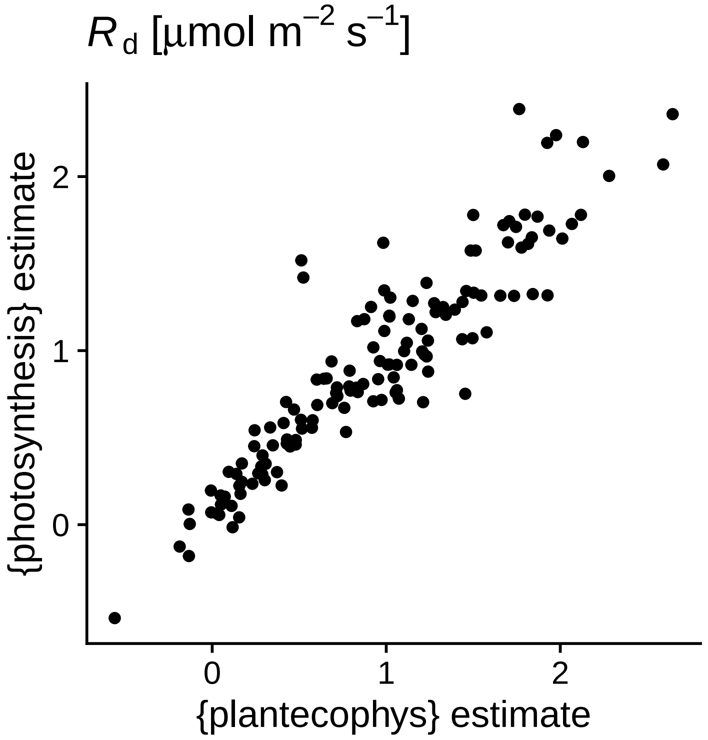

References

Farquhar, G. D., S. von Caemmerer, and J. A. Berry. 1980. "A Biochemical Model of Photosynthetic CO Assimilation in Leaves of C Species." *Planta* 149 (1): 78–90. https://doi.org/10.1007/BF00386231.

Kumarathunge, Dushan P., Belinda E. Medlyn, John E. Drake, Mark G. Tjoelker, Michael J. Aspinwall, Michael Battaglia, Francisco J. Cano, et al. 2019. "Acclimation and Adaptation Components of the Temperature Dependence of Plant Photosynthesis at the Global Scale." *New Phytologist* 222 (2): 768–84. https://doi.org/10.1111/nph.15668.

Slot, Martijn, and Klaus Winter. 2017. "In Situ Temperature Relationships of Biochemical and Stomatal Controls of Photosynthesis in Four Lowland Tropical Tree Species." *Plant, Cell & Environment* 40 (12): 3055–68. https://doi.org/10.1111/pce.13071.
