# Supplementary material for: Principles of resilient coding for plant ecophysiologists
Source: AoB Plants. 2021 Sep 19;13(5):plab059. doi: 10.1093/aobpla/plab059 (PMC8501907; doi:10.1093/aobpla/plab059)
Supplement: plab059_suppl_Supplementary_Table_S1 [file plab059_suppl_supplementary_table_s1.docx]

**Supplementary Information**

**Table S1. List of additional {photosynthesis} functions with applications and descriptions.**

| Functions | |  |
| --- | --- | --- |
| Applications | Function | Description |
| Gas Exchange | aq_response | Contains the light response model used by fit_aq_response |
| Modeling | bake-par | Checks that bake has worked |
| Modeling | bake | Temperature scales input parameters |
| Modeling | conductance | Provides calculations for leaf conductances to CO_2_ |
| Modeling | constants | Checks formatting of physical constant inputs |
| Modeling | enviro-par | Checks formatting of environmental inputs |
| Modeling | FvCB | Contains the model of Farquhar et al. (1980) for modeling |
| Gas Exchange | gs_models | Contains all stomatal conductance models that can be fit with fit_gs_model |
| Gas Exchange | j_calculations | Contains the electron transport models for fitting *A-C_i_* curves |
| Modeling | leaf-par | Ensures proper formatting of leaf parameter inputs |
| Modeling | parameter_names | Gets a vector of parameter names |
| Modeling | photosynthesis | Contains several functions necessary for photo |
| Gas Exchange | t_functions | Contains all temperature response functions that can be fit with fit_t_response |
| Modeling | utils | Contains unit conversion functions for modeling |
